# 

**Published:** 1877-04

**Authors:** 


### BOOKS AND PAMPHLETS RECEIVED.

Mothers and Daughters. Practical Studies for the Conservation of the Health of Girls. By T. S. Verdi, A.M., M.D., etc. 1877.

The Medical Men of the Revolution, with a brief history of the Medical Department of the Continental Army. Containing the Names of nearly 1200 Physicians. An Address before the Alumni Association of Jefferson Medical College, 1876. By J. M. Toner, M.D. 1876.

Remarks on Intra-Uterine Polypi, etc. By A. Reeves Jackson, A.M., M.D. 1876.

Milk-Sickness. A Paper. By W. H. Philips, M.D.

Second Annual Report of the Board of Health of Georgia. 1876.

Valedictory Address, etc., Hahnemann Medical College and Hospital. By G. A. Hall, M.D., etc. 1877.

Transactions of the Thirteenth Annual Meeting of the Ohio State Medical Society, held at Put-in-Bay. Cincinnati. 1875.

Seventh Annual Report of the New York Ohpthalmic and Aural Institute. New York. 1877.

An Essay on New South Wales, the Mother Colony of the Australias. By G. H. Reid. Sidney. 1876.

The Use of Uterine Supporters. By Clifton E. Wing, M.D. Boston.

Pneumatic Pressure and the Genu—Pectoral Posture in the Reduction of Uterine Luxations. By A. Sibley Campbell, M.D.

On the Importance of the Uterine Ebb as a Factor in Pelvic Surgery. By Horatio R. Storer, M.D. Boston.

The Relations of Medicine to Modern Unbelief. A Valedictory Address by R. O. Cowling, M. D. Louisville.

Report of the Fifth International Ophthalmological Congress held at New York, Sept. 1876.

### ANNOUNCEMENTS FOR THE MONTH.

**MONDAYS.** SOCIETIES.
*Mondays, April* 2 *and* 16.—Chicago Medical Society. Regular meetings.
*Mondays, April* 9 *and* 23.—Chicago Soc. of Phys. and Surgeons. Regular meetings.
CLINICS. *Every Monday.*
At Eye and Ear Infirmary, 2 P. M.—Prof. Holmes.
At Central Dispensary (Wood and Harrison sts).—2 P. M. *Gynecological*, Dr. Adolphus; 3 P. M. *Diseases of Children* Dr. R. S. Hall.
At Mercy Hospital—2 P. M., *Surgical*, Prof. Andrews.
At Rush College—2¼ P. M., *Medical*, Dr. Bridge.
At Chicago College, 2 P. M. *Gynecological*—Prof. Merriman.
LECTURES. *Every Monday.*
At Rush Medical College (Harrison and Wood sts.)—9 to 1 o'clock, Drs. Wadsworth, Jackson, Danforth and Knox. At Chicago College—8 to 11, Profs. Rutter, Jewell, and Curtis; 3½, Nelson.

**TUESDAYS.** SOCIETIES.
*Tuesday, April* 10.—Academy of Science. Regular meeting at 8 P. M. (263 Wabash av.)
CLINICS. *Every Tuesday.*
At Eye and Ear Infirmary—2 P. M, ,Prof. Jones.
At County Hospital—2 P. M., *Medical*. Prof. Bevan. At 3 P. M., *Surgical*, Prof. Bogue.
At Mercy Hospital, 2 P. M. *Medical*, Prof. Hollister.
At Chicago College—2 P. M., *Gynecological*, Prof. Roler.
LECTURES. *Every Tuesday.*
At Rush College—9 to 1, Drs. Owens, Bridge, Strong and Case. At Chicago College—9 to 11, Profs. Jewell and Byford.

**WEDNESDAY.** CLINICS. *Every Wednesday.*
At County Hospital—2 P. M., *Ophthalmological*, Dr. Montgomery; 3 P. M., *Gynecological*, Prof. Fitch.
At Chicago College—3 P. M., *Gynecological*, Prof. Nelson.
At Mercy Hospital—2 P. M., *Ophthalmological*, Prof. Jones.
At Central Dispensary—2 P. M., *Surgery*, Dr. Loomis; *Diseases of Chest*, Dr. Ingals; 3, *Gynecological*, Prof. Etheridge.
LECTURES. *Every Wednesday.*
At Rush College—9 to 1, Drs. Wadsworth, Ingals and Sawyer. At Chicago College—9 to 11, Profs. Hyde and Steele; 3½, Andrews.

**THURSDAYS.** CLINICS *Every Thursday.*
At Eye and Ear Infirmary—2 P. M., Prof. Hotz.
At Mercy Hospital—2 P. M., *Medical*, Prof. Davis.
At Rush College—2 P. M., *Medical*, Prof. Ross; 3 P. M., *Diseases of the Nervous System*, Prof. Lyman.
At Chicago College—2 P. M., *Gynecological*, Prof. Merriman.
At Central Dispensary—2 P. M., *Surgical*, Dr. Graham.
LECTURES. *Every Thursday.*
At Rush College—9 to 1, Drs. Hayes, Bridge, Strong and Case. At Chicago College—9 to 11, Prof. Quine and Steele; 3½, Davis or Johnson.

**FRIDAYS.** SOCIETIES.
*Friday, April* 13.—State Microscopical Society of Illinois. Regular meeting, 8 P. M.
CLINICS. *Every Friday.*
At County Hospital—2 P. M., *Medical*, Prof. Bevan; 3 P. M., *Surgical*, Prof. Bogue.
At Mercy Hospital—2 P. M., *Medical*, Prof. Davis.
At Chicago College—2 P. M., *Gynecological*, Prof. Roler.
At Central Dispensary—2 P. M., *Diseases of Chest*, Dr. Harroun; 3 P. M., *Dermatological*, Dr. Maynard.
LECTURES. *Every Friday.*
At Rush College—9 to 1, Drs. Wadsworth, Jackson, Strong and Knox. At Chicago College—8 to 11, Profs. Jones, Hyde, and Curtis; 3½, Bond.

**SATURDAYS.** CLINICS. *Every Saturday.*
At Chicago College—2 P. M., *Surgical*, Prof. Andrews or Isham; *Gynecological*, Prof. Nelson; 3 P. M., *Medical*, Prof. Johnson.
At Rush College—2 P. M., *Surgical*, Prof. Gunn.
LECTURES. *Every Saturday.*
At Rush College—9 to 1, Drs. Owens, Bridge, Sawyer and Danforth. At Chicago College—8 to 11, Profs. Merriman, Quine and Byford; 3½, Hollister.

At all the above named Clinics visiting regular practitioners are, we believe, admitted.

At the South Side Dispensary (Chicago College) there are six daily special Clinics, for sections of the classes of the Chicago College.

The Spring Course of Rush College begins March 7th. The Winter Course of the Chicago College ends March 20th.

